# Hydromorphone vs sufentanil in patient-controlled analgesia for postoperative pain management: A meta-analysis

**DOI:** 10.1097/MD.0000000000028615

**Published:** 2022-01-21

**Authors:** Zhong-Biao Nie, Zhi-Hong Li, Bin Lu, Yao-Yao Guo, Ran Zhang

**Affiliations:** a Shanxi Bethune Hospital, Pharmaceutical Department, Taiyuan, China; b Shanxi Bethune Hospital, Anesthesiology Department, Taiyuan, China; c Shanxi Bethune Hospital, Pain Department, Taiyuan, China; d Affiliated Hospital of Shanxi University of Traditional Chinese Medicine, Nephrology Department, Taiyuan, China.

**Keywords:** hydromorphone, meta-analysis, patient-controlled analgesia, sufentanil, systematic review

## Abstract

**Background:**

Patient-controlled analgesia (PCA) is an effective method of postoperative pain, there have been many studies performed that have compared the efficacy of hydromorphone with continuous sufentanil. The purpose of this systematic review is to compare the efficacy and safety of hydromorphone and sufentanil.

**Methods:**

Seven databases were searched for controlled trials to compare the efficacy and safety of hydromorphone and sufentanil. After selecting the studies, extracting the data, and assessing study quality, the meta-analysis was performed on several of the studies with RevMan 5.3.

**Results:**

Thirteen studies comprised of 812 patients were found. The pain intensity of the hydromorphone group was significantly lower than that of the sufentanil group at 12 hours. With no statistical difference at 24 to 48 hours (MD_12_ = −1.52, 95% CI [−2.13, −1.97], *P* <.05). The sedation intensity of the hydromorphone group at 12, 24, and 48 hours were lower than those of the sufentanil group, with no statistical difference (MD_12_ = −0.03, 95% CI [−0.18, 0.12], *P* > .05; MD_24_ = −0.20, 95% CI [−0.42, 0.03], *P* > .05; MD_48_ = −0.03, 95% CI [−0.18, 0.11)], *P* > .05). The PCA requests in the hydromorphone group were less than that in the sufentanil group, and there was no significant difference (RR = −0.20, 95% CI [−1.93,1.53], *P* > .05). The incidence of adverse events in the hydromorphone group was less than that in the sufentanil group, and there was a statistical difference: (RR = 0.61, 95% CI [0.47,0.79], *P* < .05).

**Conclusion:**

Compared with sufentanil, PCA with hydromorphone was more effective in relieving pain and PCA requests 12, 24, and 48 hours after operation, and significantly reduced the incidence of adverse events, but it did not have an advantage in sedation intensity.

## 1 Introduction

Postoperative pain is an acute pain that occurs immediately after surgery, usually lasting no more than 7 days. Effective postoperative analgesia not only alleviates the pain of the patient, but also helps to accelerate the recovery of the disease.^[1]^ Patient-controlled analgesia (PCA) is an effective method of perioperative analgesia, including subcutaneous PCA, epidural PCA, intravenous PCA (PCIA), and peripheral nerve block PCA. Patients can adjust the time and dose of injection of analgesics as needed to meet the analgesic requirements.^[2]^

Hydromorphone is a new kind of opioid analgesic. It has the characteristics of strong analgesic effect, long duration, and less adverse events than fentanyl. It is suitable for the treatment of postoperative acute pain, and it would provide long-acting pain relief due to its hydrophilicity and induce fewer adverse events due to its lipophilicity. It is well suited for Enhanced Recovery After Surgery protocols.^[3–7]^ Sufentanil is a potent opioid analgesic with high selectivity of mu agonists. It has a definite analgesic effect and has the characteristics of stable cardiovascular function, and it is a cheap synthetic opioid with a high therapeutic index and a quick response, is an attractive drug for postoperative pain.^[8–12]^ In recent years, many clinical studies on the efficacy and safety of these 2 drugs for PCA have been made, however, these results were controversial. Therefore, we made the systematic review and meta-analysis comparison of the effects between the 2 drugs.

## 2 Methods

Data collection and analysis were performed by the best practice Cochrane Association guidelines^[13]^ and Preferred Reporting Items for Systematic Reviews and Meta-Analyses (PRISMA) guidelines for systematic reviews.^[14]^ The ethical approval and informed consent were unnecessary since the meta-analysis was aimed to summarize the previous studies.

We sought randomized controlled trials (RCTs) that the clinical effects of hydromorphone and sufentanil for PCA after an operation. Reports were identified by Pub Med, EMBASE TM, Cochrane Central Register of Controlled Trials, and 3 Chinese databases, including the Chinese Biomedical Literature Database, China National Knowledge Infrastructure, and Wan fang Data using the following search terms as keywords and text words: “hydromorphone”, “sufentanil”, “PCA” “patient-controlled analgesia,” “self-administered,” “pain,” “analgesia,” “postoperative,” and “surgery.” Alternative spellings of the search terms were also used. Without restriction to regions, publication types, or languages confining the search to studies published between inception and July 2, 2018.

### 2.1 Inclusion and exclusion criteria

Inclusion criteria were:

1. RCTs;
2. adult surgical patients receiving postoperative PCA;
3. the use of opioid for a PCA strategy; and
4. postoperative pain-related outcomes and PCA-related adverse events.

Exclusion criteria were:

1. hydromorphone was not compared with sufentanil;
2. animal trials, reviews, and other genres, repeated publication;
3. only abstract and lack of full text, or full text does not provide sufficient raw data;
4. abstracts of scientific meetings, unpublished observations, and correspondence.

Two reviewers identified all studies that appeared to fit the inclusion criteria for the full review. Two reviewers independently selected studies for inclusion in the review. Any disagreements were referred to a third reviewer. If data were reported in a format that did not allow inclusion in the meta-analysis, we contacted the authors and asked them to release data. We identified a total of 13 RCTs. The study characteristics for each included trial are shown in Table 1.

**Table 1:** Characteristics of included trials and the PCA protocols.

				Intervention measures			
Reference	Analgesic mode	Sample size	ASA classification	hydromorphone group	sufentanil group	Loading dose/ basal infusion /bolus dose/lockout interval	Measurement index^∗^
ZF Yu 2015 I–IV^[15]^	PCSA	40/40	I–II	0.06–0.09 mg/kg	150 μg	5 mL/2 mL × h^−1^/1 mL × time^−1^/10 min	A, P
LH Liu 2015^[16]^	PCEA	35/35	I–II	150 μg	100 μg	5 mL/2 mL × h^−1^/1 mL × time^−1^/10 min	V, A, R, P
XH Sun 2015 I∼II^[17]^	PCIA	50/50	I–II	2/4 mg	50 μg	none/2 mL × h^−1^/0.5 mL × time^−1^/15min	V, A, R, P
YW Cui 2015^[18]^	PCIA	30/30	I–II	8 mg	100 μg	5 mL/4mL × h^−1^/2 mL × time^−1^/30 min	V, A, R, P
XF Cao 2017^[19]^	PCIA	30/30	I–II	120–140 μg/kg	2-3 μg/kg	none/2 mL × h^−1^/2 mL × time^−1^/15min	V, A, P
XZ Qi 2018^[20]^	PCIA	30/30	I–II	200 μg/kg	2 μg/kg	5 mL/2 mL × h^−1^/2ml × time^−1^/15min	V, A, P
ZY Zhao 2014^[21]^	PCIA	25/25	I–II	100 μg/kg	3 μg/kg	none/2 mL × h^−1^/0.5 mL × time^−1^/15 min	A, R, P
R Han 2017^[22]^	PCIA	31/31	I–II	8 mg	100 μg	-	V, P
HG Wang 2014^[23]^	PCIA	30/30	I–II	96 μg/kg	2.4 μg/kg	2(0.15)^∗∗^μg × kg^−1^/2 mL × time^−1^/2 mL × time^−1^/20 min	V, R, P
MX Su 2017^[24]^	PCEA	30/30	I–II	7 mg	150 μg	5 mL/2 mL × h^−1^/2 mL × time^−1^/30 min	V, P
FM Yang 2016^[25]^	PCIA	40/40	I–II	10 mg	100 μg	none/2 mL × h^−1^/2 mL × time^−1^/20min	V, R, P
XC Bian 2017 I–III^[26]^	PCIA	20/20	I–II	50–150 μg/kg	100 μg	none/2 mL × h^−1^/0.5 mL × time^−1^/15 min	V, R, P
Y Tao 2015 I–II^[27]^	PCEA	20/20	II–III	25/50 μg/kg	1 μg/kg	none/2 mL × h^−1^/0.5mL × time^−1^/15min	V, A, R, P

### 2.2 Data extraction

All studies comparing hydromorphone to sufentanil for PCA were included. The outcome were: pain intensity, as measured by the Visual Analogue Scale (VAS) score, VAS is the most common measurement to assess pain intensity. It is scored on a range of either 0 to 10(0 = no pain, 10 = worst pain). Sedation intensity, as measured by the Ramsay score at the 12/24/48 hours after operation; PCA requests for analgesia, adverse events of patients. We also conducted subgroup analyses to explore the various types of PCAs on the incidence of postoperative pain management. We separated PCIA and epidural PCA for analysis.

### 2.3 Statistical analyses

Results that were pooled from the included studies were meta-analyzed. For continuous data, a Mantel–Haenszel Chi-Squared test was used and expressed as the mean difference with 95% CI, and for dichotomous data an inverse variance was used and expressed as risk ratio with 95% CI. In both cases *P* < .05 was considered significant. Heterogeneity was analyzed using a Chi-Squared test on N-1 degrees of freedom, with an alpha of 0.05 used for statistical significance and with the *I*^2^ test. *I*^2^ values of 25%, 50%, and 75% correspond to low, medium, and high levels of heterogeneity. A fixed-effect model was used unless statistically significant high heterogeneity (*I*^2^ > 75% was considered as significantly high heterogeneity) existed between studies. A random-effects model was employed if heterogeneity existed. An assessment of the methodological quality of the included studies into the meta-analysis was conducted in line with the Cochrane handbook.^[28,29]^ Review Manager (Rev Man 5.3) was used to plot the quality assessment.

## 3 Results

### 3.1 Study selection

**Figure 1:**
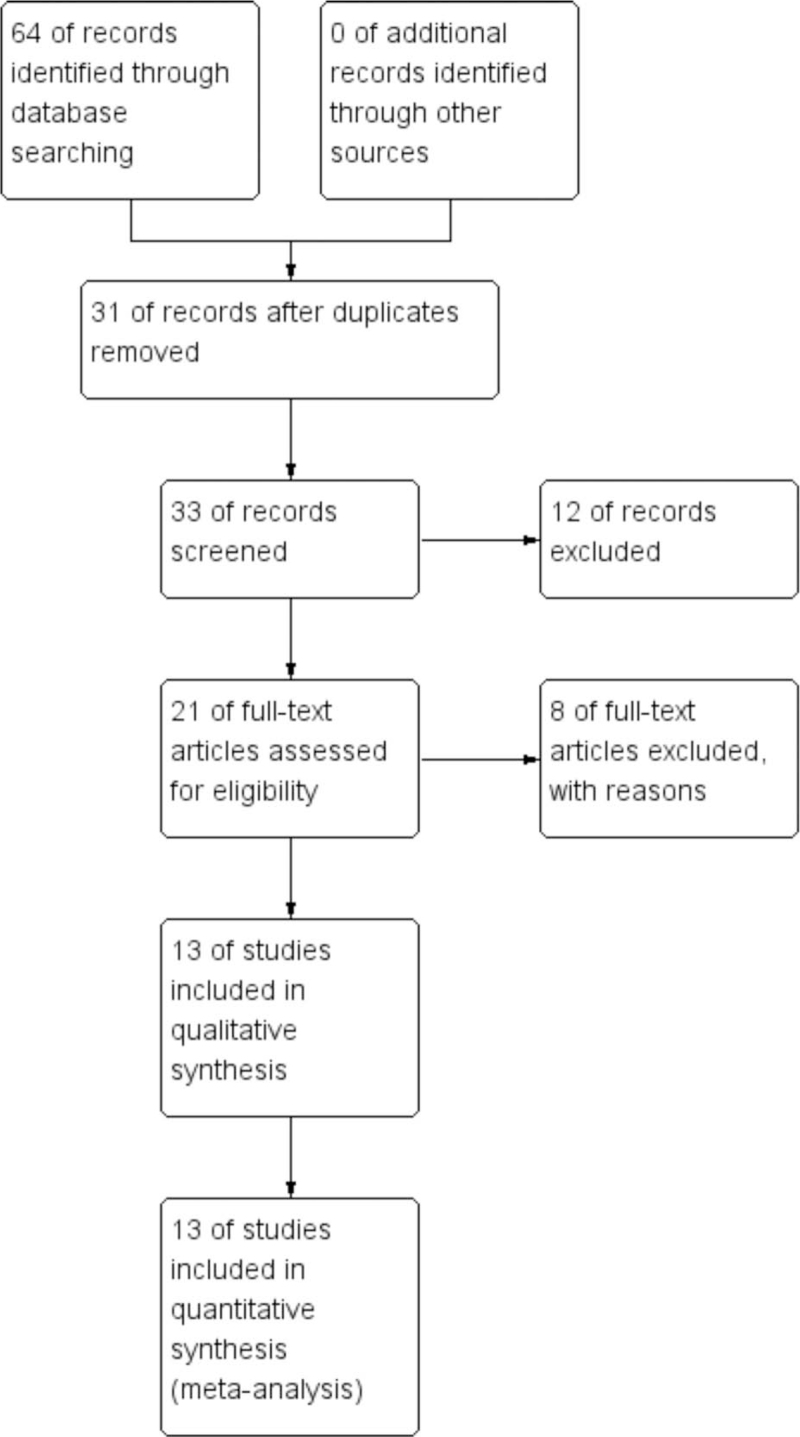
Flow diagram of study selection and identification.

A total of 64 citations were identified for eligibility through the systematic literature search. After exclusion of duplicate publications and full-text review of the relevant studies. A total of 13 cohort studies encompassing 812(411 patients were hydromorphone group and 401 patients were sufentanil group) individuals were included in the quantitative synthesis (Fig. 1). All the included studies were randomized controlled trials. All of the studies were from centers in China and all studies were single-center studies.

### 3.2 Risk of bias assessment

**Figure 2:**
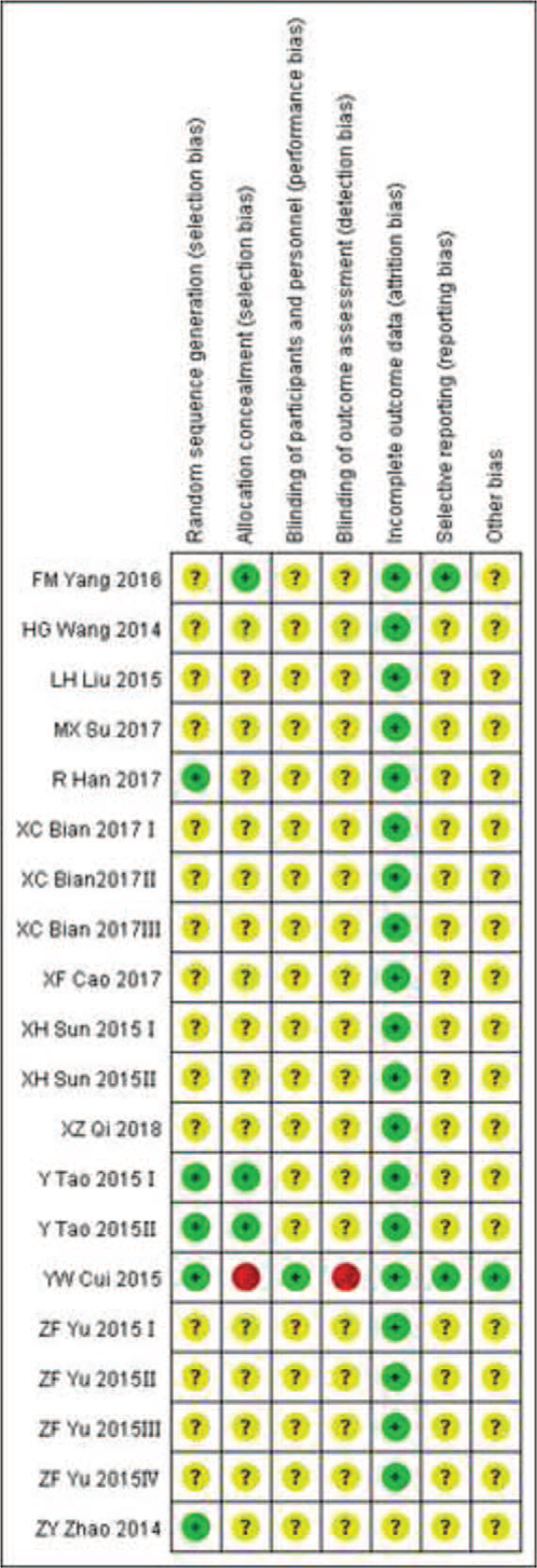
Risk of bias graph for included studies.

The details of methodologic quality are shown in Figure 2. Ten studies^[15–17,19–21,23–26]^ did not describe the details of random sequence generation. All studies had unclear risks of bias due to blinding of outcome assessment, incomplete outcome data, and selective reporting.

### 3.3 Meta-analysis results

#### 3.3.1 Postoperative pain intensity

**Figure 3:**
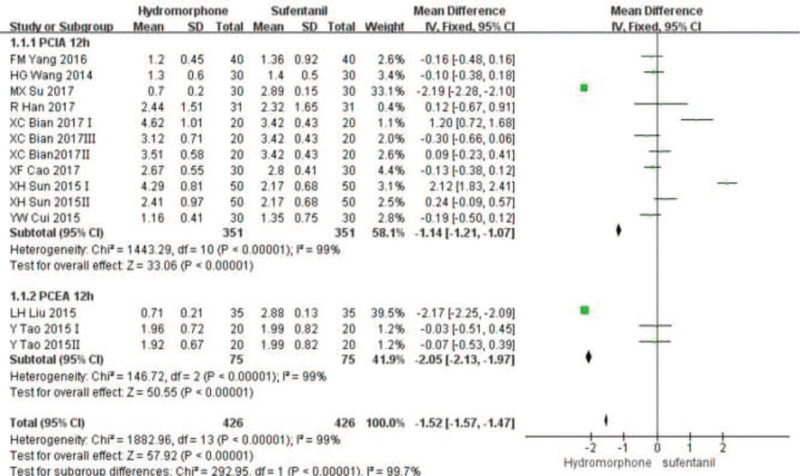
The pain intensity analysis of hydromorphone vs sufentanil for PCA up to postoperative 12 hours.

**Figure 4:**
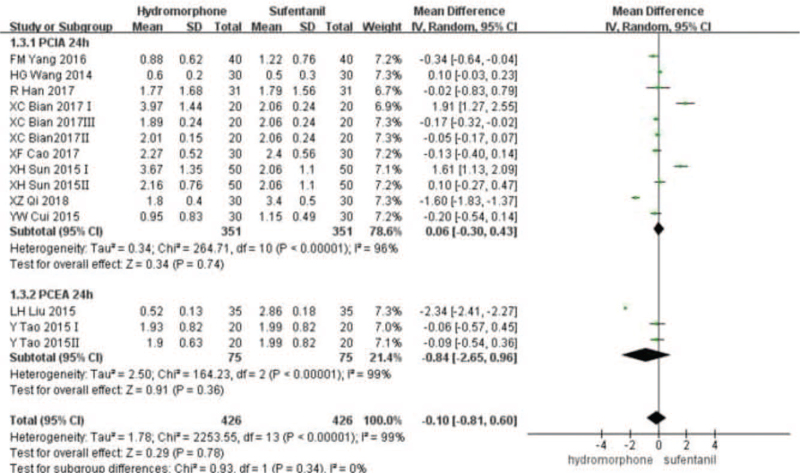
The pain intensity analysis of hydromorphone vs sufentanil for PCA up to postoperative 24 hours.

**Figure 5:**
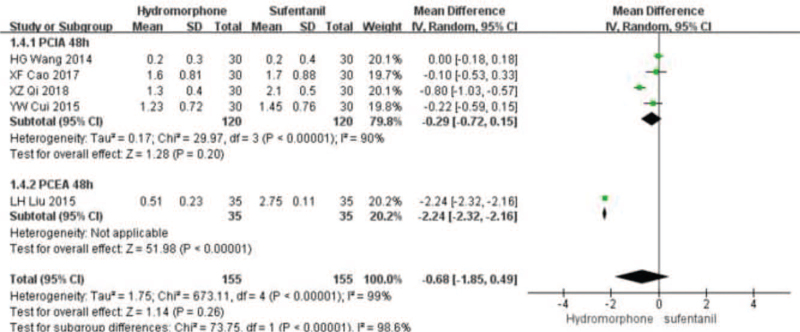
The pain intensity analysis of hydromorphone versus sufentanil for PCA up to postoperative 48 hours.

Eleven studies^[16–20,22–27]^ provided VAS score data for patients at different time points 12, 24, and 48 hours after surgery. Statistical heterogeneity was found among the studies (*P* < .001, *I*^2^ = 99%). A random-effect model was used to analyze the data. Meta-analysis showed that pain score of the hydromorphone group was significantly lower than that of the sufentanil group at 12 hours. With no statistical difference at 24 to 48 hours (Weighted mean difference [MD] was MD_12_ = −1.52, 95% CI [−2.13, −1.97], *P* < .05; MD_24_ = −0.10, 95% CI [−0.81, 0.06], *P* > .05; MD_48_ = −0.68, 95% CI [−1.85, 0.49], *P* > .05), see Figures 3–5.

#### 3.3.2 Postoperative sedation intensity

**Figure 6:**
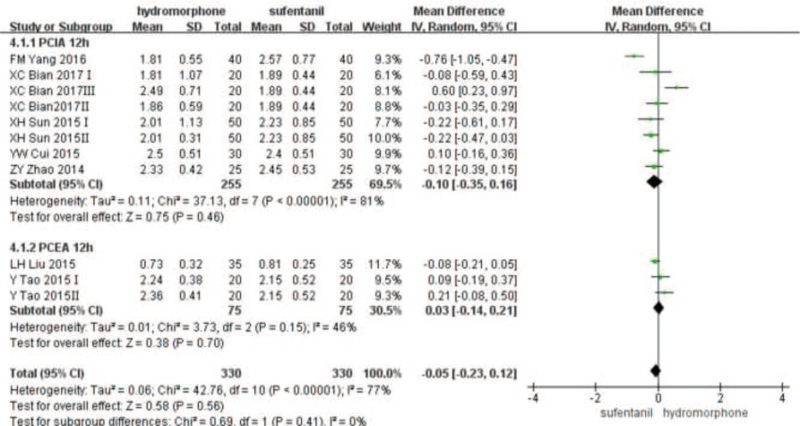
The Ramsay intensity analysis of hydromorphone versus sufentanil for PCA up to postoperative 12 hours.

**Figure 7:**
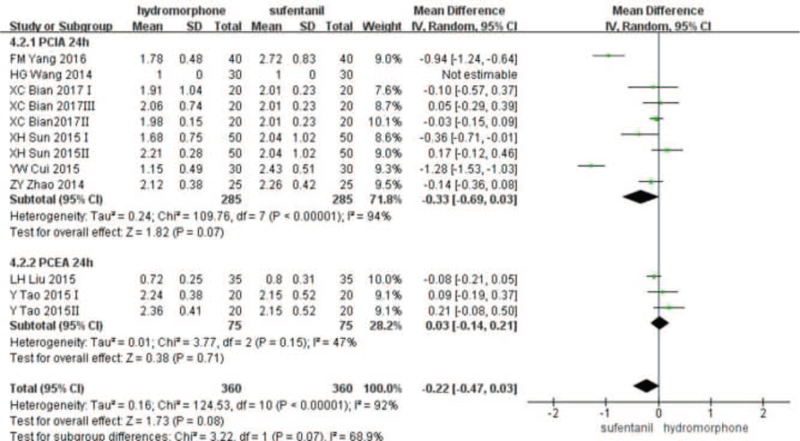
The Ramsay intensity analysis of hydromorphone versus sufentanil for PCA up to postoperative 24 hours.

Eight studies^[16–18,21,23,25–27]^ provided Ramsay score data for patients at different time points 12 to 24 hours after surgery. Statistical heterogeneity was found among the studies (*P* < .001, *I*^2^ = 75%; 90%; 83%). Random effect model was used to analyze the data. Meta-analysis showed that the sedation scores of the hydromorphone group at 12 to 24 hours were lower than those of the sufentanil group, with no statistical difference. (MD_12_ = −0.05, 95% CI [−0.23, 0.12], *P* > .05; MD_24_ = −0.22, 95% CI [−0.47, 0.03], *P* > .05), see the Figures 6 and 7.

#### 3.3.3 PCA requests for analgesia

**Figure 8:**
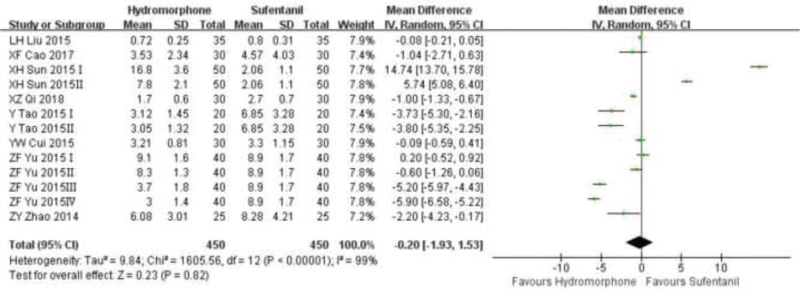
The PCA requests analysis of hydromorphone versus sufentanil for PCA up to postoperative 12/24/48 hours.

Eight studies^[15–21,27]^ reported the number of PCA requests. Statistical heterogeneity (*P* < .001, *I*^2^ = 99%) was found among the studies. Random effect model was used to analyze the results. Meta-analysis showed that the number of PCA requests in the hydromorphone group was less than that in the sufentanil group, and there was no significant difference (RR = −0.20, 95% CI [−1.93,1.53], *P* > .05), see the Figure 8.

#### 3.3.4 Postoperative adverse reaction rate

**Figure 9:**
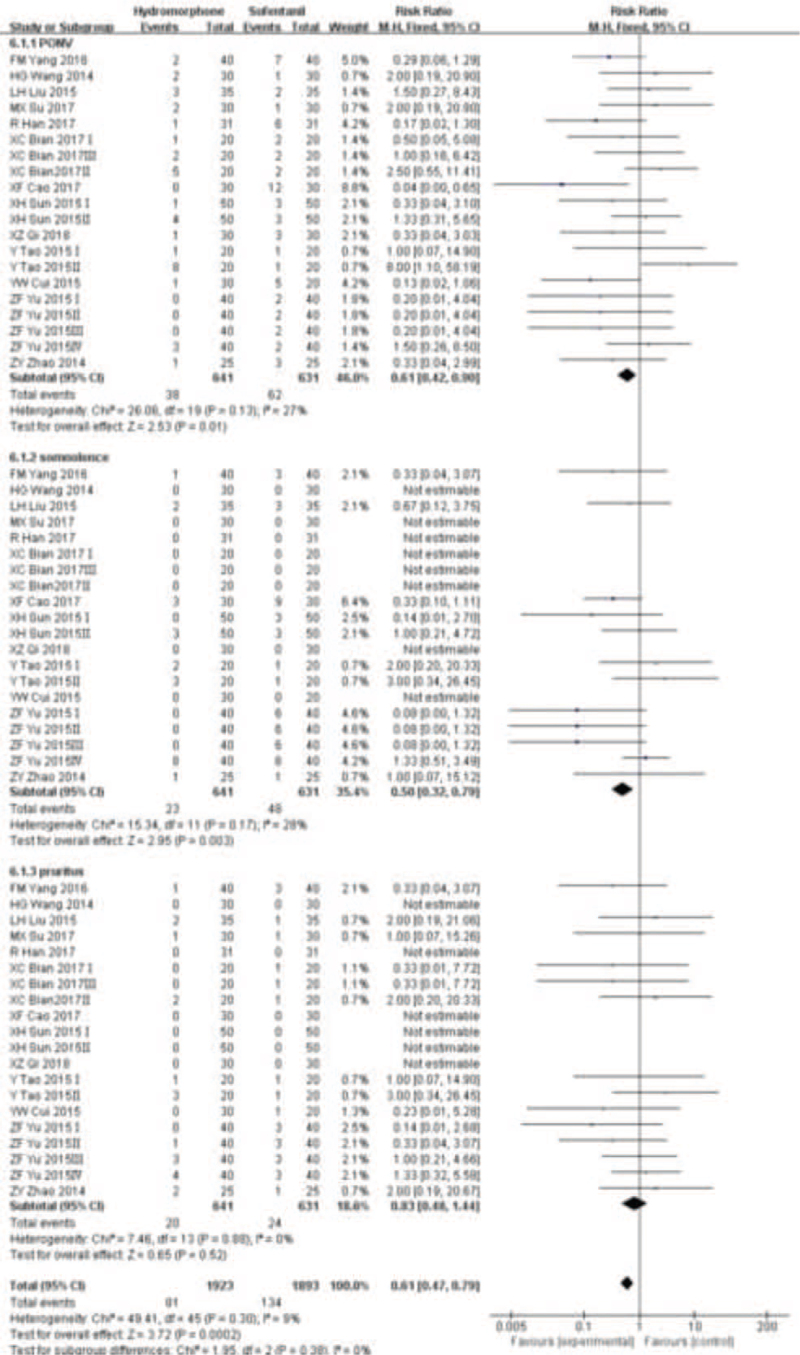
The adverse events analysis of hydromorphone versus sufentanil for PCA up to postoperative 12/24/48 hours.

Thirteen studies^[15–27]^ reported the incidence of postoperative adverse events. The main adverse events include postoperative nausea vomiting (PONV), somnolence, pruritus. Therefore, we set up 3 subgroups for analysis. Statistical heterogeneity was found among the studies (*P* < .001, *I*^2^ = 9%). Random effect model was used to analyze the incidence of postoperative adverse events. Meta-analysis showed that the incidence of adverse events in the hydromorphone group was less than that in the sufentanil group, and there was a statistical difference (RR = 0.61, 95% CI [0.47,0.79], *P* < .05), see Figure 9.

#### 3.3.5 Publication bias

**Figure 10:**
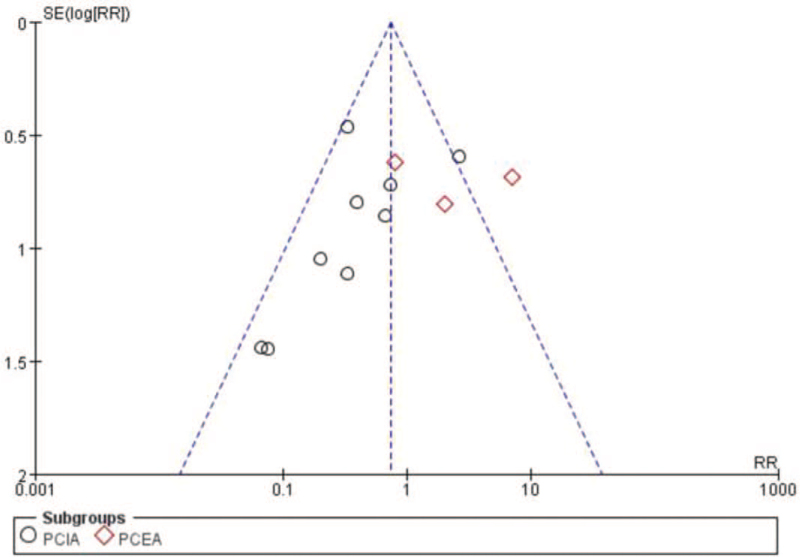
Assessment of publication bias using a funnel plot. The plot shows the absence of publication bias.

The funnel plot was used to analyze the incidence of adverse events. As a result, the distribution of the inverted funnel plot is asymmetrical, suggesting that there may be large publication bias and clinical heterogeneity in the included literature, see Figure 10.

## 4 Discussion

Patient-controlled analgesia pump, which is characterized by high efficiency, no blind zone for analgesia, and stable blood concentration, has been widely used in postoperative rapid analgesia. At present, sufentanil is the representative used in intravenous analgesia in a clinic,^[2,30]^ characterized by obvious analgesic effect and long duration of action. However, it always causes vertigo, pruritus, nausea, and vomiting. Hydromorphone, as a novel powerful opioid with a clear analgesic effect, has been reported both at home and abroad.^[7]^ A meta-analysis of Felden ^[4]^ has shown that the clinical efficacy of hydromorphone in acute pain is slightly superior to morphine. There are many studies on the clinical efficacy of hydromorphone and sufentanil in PCA, but evidence-based medicine is lacking for their efficacy and safety.

Our meta-analysis showed that compared with sufentanil, hydromorphone can significantly reduce postoperative pain for 12 hours, there was no significant difference in sedation, and PCA requests, but the incidence of adverse events in the hydromorphone group was significantly better than that in the sufentanil group, especially in PONV and somnolence. It may be due to the hydrophilicity of hydromorphone, which can provide long-lasting analgesic effects and cause fewer adverse events.^[4,32]^

Although some studies have found that basal infusion of sufentanil PCIA can effectively relieve pain with few adverse events, ^[33]^ American Pain Society recommends against routine basal infusion of opioids with i.v. PCA in opioid-naive adults. ^[34]^ Therefore, adverse events such as PONV in the included studies may be related to the basal infusion.

Our study revealed an article by Hua,^[31]^ in which the postoperative VAS score was higher than 10 (the highest score is 10). Therefore, we contacted the author but did not receive any reply, so we excluded this article.

There are multiple-dose comparative studies in some articles.^[15,17,26,27]^ We analyzed separate experiments with different doses.

There are shortcomings and limitations in this study:

1. This systematic review included 13 studies, all of which were Chinese articles. Although we searched the Cochrane Central Register of Controlled Trials, MEDLINE, EMBASE, and WHO International Clinical Trials Registry Platform, we found few English language studies.
2. There were some differences in the dosage of each study, and the loading dose/basal infusion/locking time of PCA drugs included in the study were also different.

The literature reveals that, between 1996 and 1999, 25 sedation assessment tools were published, of which 3 have been rigorously tested for validity and reliability in adults: the motor activity assessment scale, the Ramsay sedation scale and the sedation agitation scale (SAS) Their study can be criticized, however, only the Ramsay scale had been validated adequately for use in the critical care environment, so more studies have chosen Ramsay scale.^[35]^

## 5 Conclusion

The results demonstrate that compared with sufentanil, PCA with hydromorphone is more effective in relieving postoperative pain at 12, 24, and 48 hours and reducing PCA request, and significantly decreases the incidence of postoperative adverse events. However, its effect on analgesia is not obvious. The quality of clinical studies is relatively low and the dosage in most studies is different; therefore, high-quality multicenter, randomized, parallel-controlled, and blind trials are needed for further study. The studies are of low quality and are all of Chinese origin, so this meta-analysis conclusion is only suitable for Chinese.

## Author contributions

**Conceptualization:** Zhongbiao Nie, Bin Lu, Yao-yao Guo.

**Data curation:** Zhongbiao Nie, Zhi-Hong Li, Bin Lu, Yao-yao Guo, Ran Zhang.

**Formal analysis:** Zhongbiao Nie, Bin Lu.

**Methodology:** Zhongbiao Nie, Zhi-Hong Li, Bin Lu, Ran Zhang.

**Software:** Zhongbiao Nie, Zhi-Hong Li, Ran Zhang.

**Writing – original draft:** Zhongbiao Nie, Zhi-Hong Li, Ran Zhang.

**Writing – review & editing:** Zhongbiao Nie, Zhi-Hong Li, Ran Zhang.
